# Asparaginase with Combined Mutations: Optimized Biochemistry and Lowered Allergic Risk

**DOI:** 10.1021/acsptsci.5c00547

**Published:** 2025-11-06

**Authors:** Tales Costa-Silva, Grace V. Ruiz-Lara, Iris Munhoz Costa, Adalberto Pessoa, Gisele Monteiro

**Affiliations:** † Center for Natural and Human Sciences, Federal University of ABC, Santo André, São Paulo 09210580, Brazil; ‡ Department of Biochemical and Pharmaceutical Technology, School of Pharmaceutical Science, University of São Paulo, São Paulo SP 05508-000, Brazil

**Keywords:** asparaginase, biologics, biobetter, allergy, ALL-molecular diagnosis and therapy, ALL-molecular biology

## Abstract

Biotechnology and biomedical advances have driven the development of novel biopharmaceuticals to meet growing clinical demands. Among approved biologics, native *Escherichia coli* asparaginase has been under continuous optimization to improve thermostability, half-life, resistance to human proteases, and reduce adverse effects, particularly allergenicity. Here, we engineered an antileukemic biobetter by combining the substitutions P40S/S206C—previously identified by our group as less immunogenic and with extended bloodstream activity in mice—with N24S, reported to enhance in vitro stability. The purified triple mutant enzyme was biochemically characterized, and its cytotoxicity against leukemic cell lines and antigenic properties in Balb/c SPF mice were evaluated. TM displayed robust asparaginase activity, a >3-fold reduction in *K*_M_ for asparagine, superior thermostability, enhanced proteolytic resistance, and a lower in silico immunogenicity score compared to wild-type. In vivo, compared to wild-type, TM showed no apparent toxicity, a lower decrease in platelet counts, reduced induction of antiasparaginase IgE antibodies, and a preserved pharmacokinetic profile. In conclusion, combined mutations conferred substantial biochemical and immunological improvements, supporting the strategy of targeted amino acid substitutions to advance next-generation asparaginase biopharmaceuticals.

## Graphical abstract

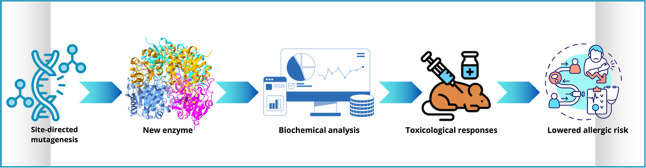

Biopharmaceuticals are a rapidly expanding class of therapeutic agents, with the global market estimated at USD 40.1 billion in 2024 and projected to grow by 11.1% annually through 2030.^1^ Among them, recombinant proteins and enzymes play a central role, particularly in oncology, autoimmune diseases, and metabolic disorders. While North America leads biopharmaceutical R&D, Latin American countries such as Brazil and Argentina are emerging players in the development and adoption of biologics and biosimilars.

Recombinant asparaginase from *Escherichia coli* (EcA II) has been a cornerstone in the treatment of acute lymphoblastic leukemia (ALL) and lymphoma since 1978. However, its clinical use is challenged by limited thermostability, short serum half-life, susceptibility to human proteases, and frequent hypersensitivity reactions.^2^ Clinically, patients treated with commercially available ASNases face adverse reactions due to enzyme short serum half-life, low stability and repeated doses to achieve antitumor effectiveness.^3^ Proteolysis of ASNase can be considered one the main mechanism of resistance in aggressive leukemias with poor outcomes, such us Philadelphia-positive (Ph+) and iAMP21 which involve AEP- and CTSB-expressing cells.^4–6^ In addition, CTSB- expressing macrophages in the tumor microenvironment also contribute to ASNase degradation and clearance,^7^ ultimately resulting in reduced responsiveness to treatment.

Protease sensitivity may also be associated with the development of anti-EcA II antibodies, as proteolytic fragments can expose cryptic epitopes that stimulate immune responses, leading either to hypersensitive reactions or to silent inactivation.^8–10^ Alternative sources, such as *Erwinia chrysanthemi* asparaginase (Erwinaze, FDA-approved in 2011), have been used in patients who develop silent inactivation or allergic reactions to EcA II, owing to the absence of immunologic cross-reactivity.^11^ Nevertheless, bioprospecting for novel ASNase sources is costly and time-consuming, which has prompted increasing interest in biobetters - engineered variants of existing enzymes with optimized properties. By enhancing substrate affinity, thermal stability or proteolytic resistance, biobetters hold the potential to improve therapeutic efficacy while lowering immunogenicity, ultimately benefiting patient outcomes.

Currently, two EcA II-based formulations are available: native EcA II (no longer available in North America) and PEGylated EcA II (Oncaspar, FDA-approved in 1994), in which the enzyme is covalently conjugated to polyethylene glycol (PEG) through a succinimidyl succinate linker. PEGylation prolongs half-life by increasing hydrophilicity and reducing both aggregation and proteolysis.^2^ Although PEG-asparaginase shows lower hypersensitivity rates compared with native EcA II,^12–15^ recent evidence has revealed the immunogenicity of PEG itself, including the formation of anti-PEG and antilinker antibodies,^16^ as well as cross-reactivity in patients previously exposed to native EcA II.^17^ To overcome these limitations, a more stable PEGylated EcA II (Asparlas, FDA-approved in 2018) was developed, employing a succinimidyl carbonate linker that improves formulation stability.^18^ In parallel, Rylaze—a recombinant *E. chrysanthemi* asparaginase expressed in *Pseudomonas fluorescens*—was approved to address supply shortages, while maintaining activity and immunogenicity profiles equivalent to Erwinaze.^11^

Therefore, the development of ASNase biobetters represent a promising strategy to deliver improved enzymes to the market, combining suitable catalytic activity and cytotoxicity with reduced or absent side effects. Brumano et al. (2019) reported several engineered variants with distinct outcomes, including partial or complete modulation of ASNase activity, reduction of glutaminase (GLNase) activity, enhanced thermal stability, increased proteases resistance, and decreased immunogenicity.^2^ Nevertheless, despite these advances, only a few candidates have undergone both comprehensive biochemical characterization and multiparametric in vivo evaluation.

Previously, our group developed the double mutant P40S/S206C, which showed improved resistance to human lysosomal proteases and reduced production of antiasparaginase antibodies in vivo, while retaining cytotoxic activity against human leukemic cell lines.^10^ In parallel, Maggi et al. (2017) reported the N24S mutant with superior thermal and storage stability and enhanced antileukemic activity in vitro.^8^ Structural analyses confirmed that both P40S/S206C and N24S adopt a tetrameric assembly with molecular weight equivalent to the wild-type (WT) enzyme. Importantly, both mutants display catalytic activity and antitumor effects comparable to the marketed PEGylated EcA II preparation.^8,10^

In this work we aimed to develop a biobetter ASNase by combining two previously characterized mutants, P40S/S206C and N24S. Our rationale was based on the observation that P40S/S206C retained only ∼60% of WT catalytic activity, while N24S maintained full activity. In addition, we sought to merge the favorable in vivo^10^ and in vitro^8^ stability profiles observed for each variant. We therefore hypothesized that combining these mutations could generate a synergistic effect on the biochemical properties and nonclinical performance of the resulting triple mutant (TM: N24S/P40S/S206C). Here, we present the biochemical characterization, in vitro cytotoxicity assays, and in vivo studies of the TM, which demonstrated enhanced substrate affinity, catalytic efficiency, stability, reduced allergenic potential, improved pharmacokinetic profile, and a lower in silico epitope score.

## Results and Discussion

Bacterial culture and recombinant expression of the *ansB* gene encoding for WT and TM enzymes were successfully carried out, yielding the expected protein production. Purity levels ≥95% were confirmed and considered adequate for subsequent analyses. The results are detailed below.

### Biochemical Characterization of Enzymes

The ASNase and GLNase activities of WT and TM are summarized in Table 1. WT exhibited higher activities, with 74.2 U/mg for ASNase and 1.85 U/mg for GLNase, compared with 52.5 U/mg and 1.2 U/mg, respectively, for TM. Thus, the relative secondary GLNase activity of TM was slightly lower than that of WT, a feature that may be advantageous in reducing glutamine-related toxicity while maintaining asparaginase activity.

**Table 1:** Specific ASNase and GLNase Activities, and Relative GLNase Activity of WT and TM Enzymes^b^

enzyme	ASNase activity (U/mg)	GLNase activity (U/mg)	Relative GLNase activity (%)
WT	74.21 ± 4.94	1.85 ± 0.08	2.49
TM	52.61 ± 3.32^a^	1.22 ± 0.04^a^	2.31

a *p* < 0.05 indicates a statistically significant difference compared with WT.

b Data are expressed as mean ± SD (*n* = 3).

Kinetic parameters and profiles are shown in Figure 1. The Michaelis–Menten constant (*K*_M_) of TM was significantly lower than that of WT (61.1 μM vs 223.7 μM, respectively). This study aimed to generate an improved ASNase variant for safer and more effective ALL therapy. The TM was rationally designed by combining a previously reported double mutant with an additional substitution near the active site. Mutations located within ∼5–10 Å of substrate-binding or catalytic sites are known to enhance substrate affinity and enantioselectivity,^19,20^ which supports the superior affinity and catalytic efficiency observed for TM compared with WT.

**Figure 1:**
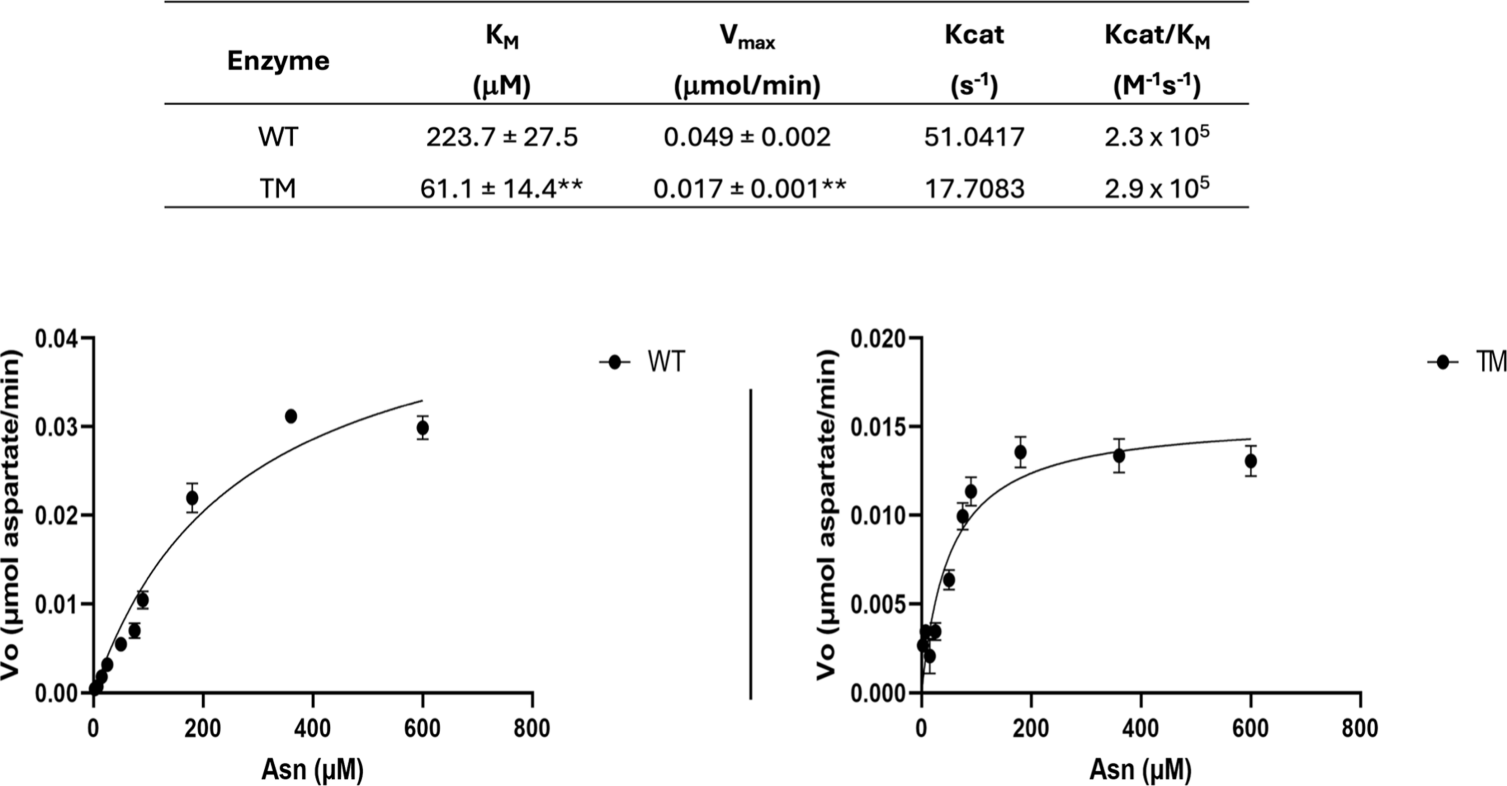
Kinetic parameters of WT and TM enzymes. **p < 0.005 indicates a statistically significant difference compared with WT. Kinetic profiles were determined using a coupled enzymatic assay with glutamic-oxaloacetic transaminase and malic dehydrogenase. To evaluate protocol variability, three independent technical replicates were performed. In addition, two analytical replicates were carried out to confirm the accuracy of the measurement results.

The optimal pH was assessed across a broad range (4.5–10.5) (Figure 2A). Both enzymes displayed maximal ASNase activity near physiological blood pH, with peaks at pH 7.5 (81.8 U/mg for WT and 72.2 U/mg for TM), and minimal activity at pH 10.5 (28.8 U/mg and 23.1 U/mg, respectively). The optimal temperature for WT and TM was 55 °C, corresponding to activities of 117.6 U/mg and 86.5 U/mg, respectively (Figure 2B). At extreme conditions, WT showed the lowest activity at 70 °C (51.4 U/mg), whereas TM was least active at 30 °C (34.6 U/mg). Importantly, at 37 °C (human body temperature), WT retained ∼70% of its maximal activity (82.4 U/mg), while TM preserved ∼83.5% (72.2 U/mg), underscoring the favorable stability of TM under physiologically relevant conditions.

**Figure 2:**
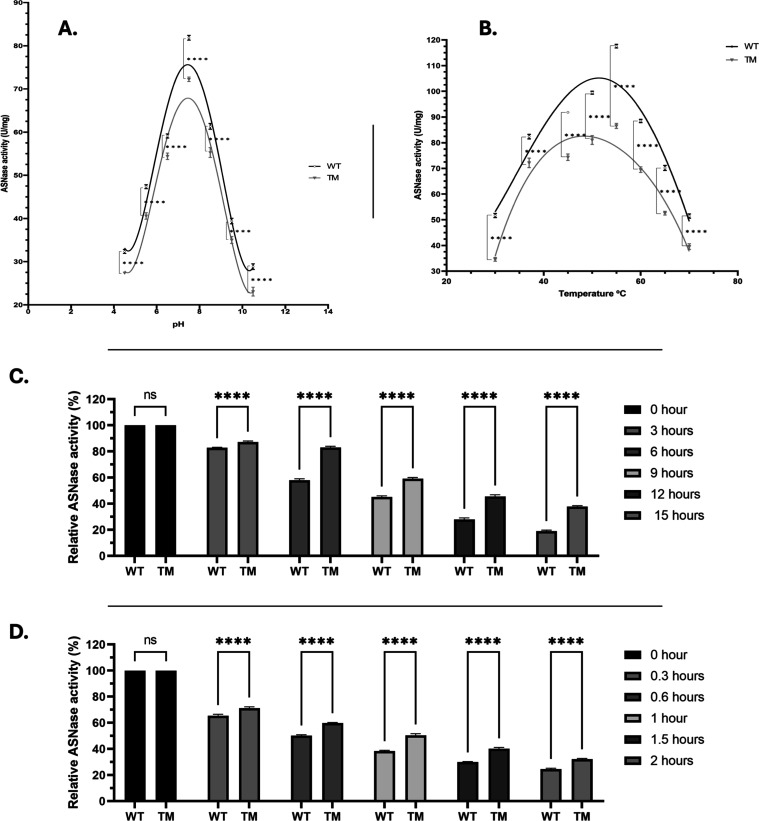
(A) ASNase activity of WT and TM at different pH values (*n* = 3). (B) ASNase activity of WT and TM at different temperatures (*n* = 3). (C) Residual ASNase activity of WT and TM at multiple time points at 37 °C, and (D) at 50 °C (*n* = 3). ****p < 0.00005 indicates a statistically significant difference compared with WT.

Thermostability was evaluated at 37 and 50 °C for both enzymes (Figure 2C,D). ASNase activity was monitored over time—at 0, 3, 6, 9, 12, and 15 h at 37 °C, and at 0, 0.3, 0.6, 1, 1.5, and 2 h at 50 °C. Overall, TM displayed greater residual activity than WT under both conditions. After 15 h at 37 °C, TM retained 38% of its initial activity, compared with 19% for WT. Likewise, after 2 h at 50 °C, TM preserved 32% of its activity, while WT retained 25%. To further assess thermal effects, the deactivation constant (*K*_d_), half-life (*t*_1/2_), and thermal stability factor (TSF) were calculated at both temperatures (Table 2).

**Table 2:** Thermal Deactivation Constant (K_d_), Half-Life (*t*_1/2_), and Thermal Stability Factor (TSF) of WT and TM Enzymes at 37 and 50 °C

temperature	37 °C		50 °C	
enzyme	WT	TM	WT	TM
*K*_d_ (hours^–1^)	0.100	0.065	1.033	0.534
*t*_1/2_ (hours)	6.93	10.66	0.67	1.30
TSF		1.54		1.94

For all parameters evaluated, the TM enzyme outperformed WT, demonstrating the positive impact of the introduced mutations. In pharmaceutical development, improved thermal stability provides significant advantages for formulation, storage, and distribution. Although lyophilization is still the most common preservation method, spray drying has emerged as an attractive alternative for large-scale manufacturing, offering a one-step, cost-effective, and highly controllable process.^21,22^ The robust thermal profile of TM indicates that it could withstand such industrial conditions, reinforcing its potential for scalable production.

Proteolytic resistance was greater in TM than in WT when challenged with the human proteases CTSB and AEP (Figure 3). After CTSB exposure (Figure 3A), TM retained 35 U/mg of ASNase activity (50% of its initial activity), whereas WT retained 30 U/mg (38%). Similarly, following AEP treatment (Figure 3B), TM preserved 38 U/mg of activity, while WT retained only 21 U/mg (54% and 27% of their initial activities, respectively). Negative controls confirmed that both enzymes maintained 100% of their original activity under identical conditions in the absence of proteases.

**Figure 3:**
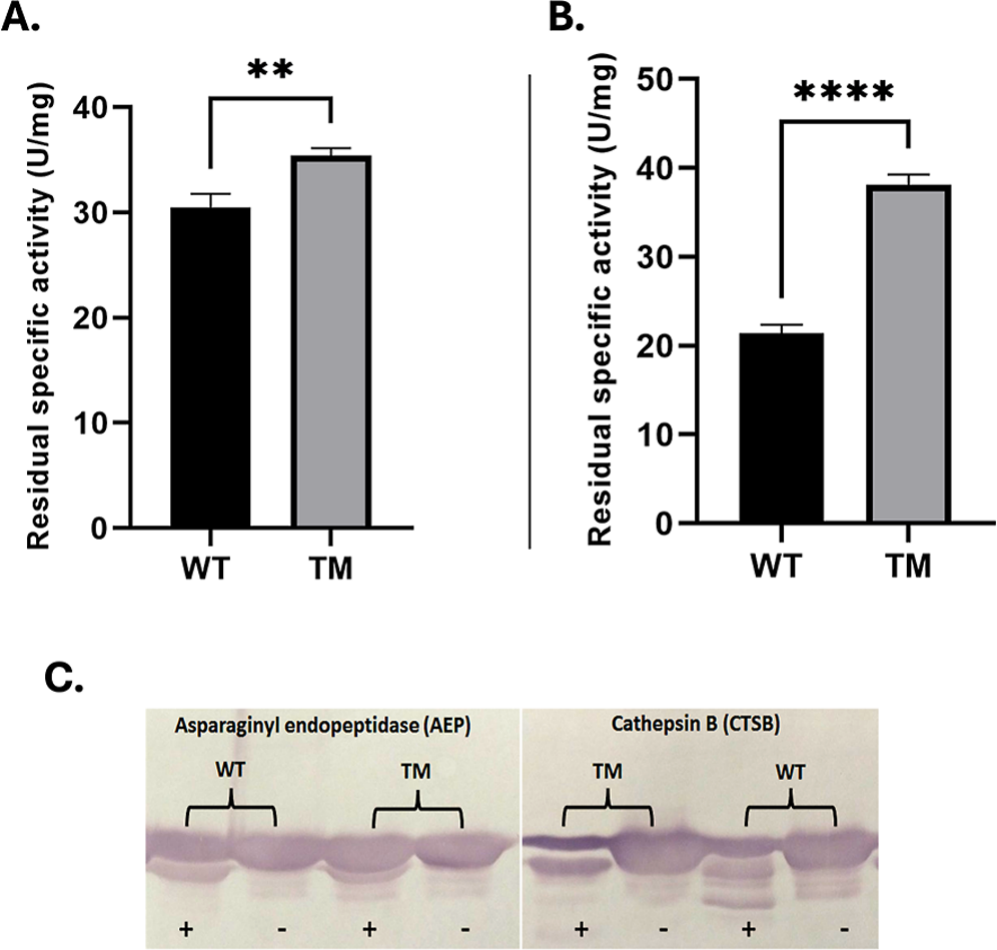
Residual ASNase activity of WT and TM after treatment with (A) CTSB and (B) AEP (*n* = 3). **p < 0.005; ****p < 0.00005 indicate statistically significant differences compared with WT. The residual activity was compared to activity before addition of proteases, as well as ASNases incubated without addition of proteases (C) Representative SDS-PAGE analysis under reducing conditions of the samples shown in (A,B).

Beyond catalytic improvements, TM exhibited markedly enhanced resistance to proteolysis by CTSB and AEP (Figure 3C)—two proteases implicated in ASNase clearance and treatment resistance in aggressive leukemia subtypes^4–6^ —as well as superior thermostability. Previous studies demonstrated that the N24S mutation increases melting temperature, stabilizes hydrogen bonding at the active site, and reduces conformational flexibility, thereby contributing to thermal and proteolytic stability.^8^ Our findings confirm these protective effects and further highlight TM as a promising biobetter candidate.

### In Silico Analysis

Predicted linear B-cell epitopes of WT and TM, together with their respective protein sequences, are shown in Figure 4. The WT enzyme displayed an average epitope score of 0.34, whereas TM showed a reduced score of 0.315. Figure 4 also illustrates the graphical epitope profiles: yellow regions indicate predicted epitopes above the threshold, while the *X*-axis corresponds to amino acid positions. The N24S substitution did not markedly affect the immunogenic potential in its region; however, the P40S and S206C mutations appear to decrease local epitope propensity, as reflected by expanded green (nonepitope) areas.

**Figure 4:**
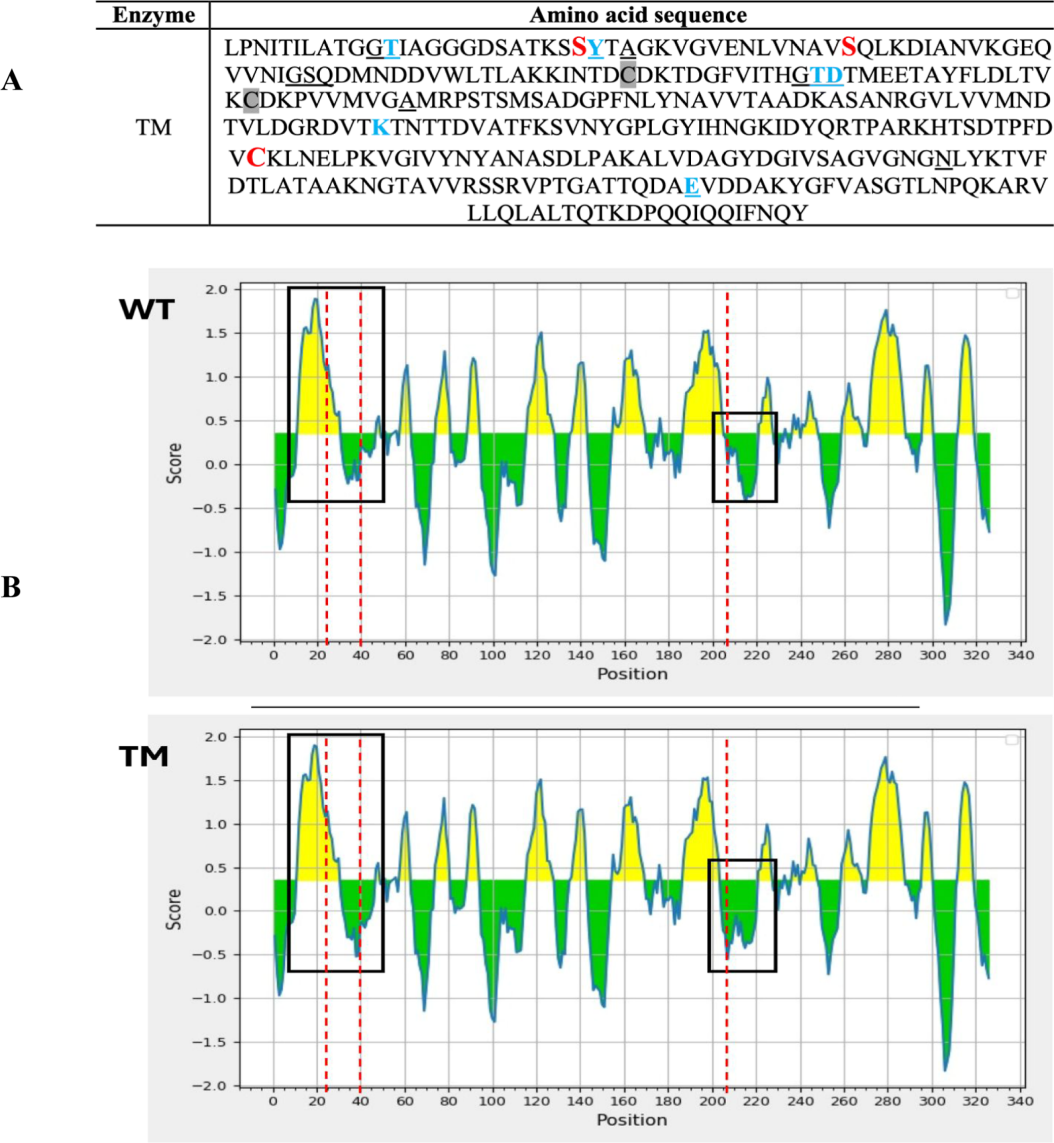
Mature amino acid sequence of the TM enzyme and its main structural features. (A) Mutations are indicated in red, the active site in blue, the aspartic acid binding site is underlined, and the disulfide bridge is highlighted. (Source: PDB and EMBL-EBI PDBsum—3ECA). (B) Predicted linear B-cell epitope profiles of WT and TM. Red dotted lines mark the positions of amino acids 24, 40, and 206, and the black box highlights the surrounding regions.

### Nonclinical Test

The MTT assay performed prior to in vivo studies showed that the TM enzyme retained strong cytotoxic potential despite the introduced mutations, with no statistically significant difference compared with WT (Table 3).

**Table 3:** IC_50_ Values from the MTT Assay in MOLT-4 and REH Leukemic Cell Lines After 72 h of Treatment with WT and TM Enzymes (*n* = 2)

	IC_50_ (U/mL)	
cell line	WT	TM
MOLT-4	0.112 ± 0.03	0.050 ± 0.03
REH	0.093 ± 0.01	0.139 ± 0.02

### Toxicological Responses In Vivo

Mice treated with both ASNases were 6–8 week-old, with an average body weight of 20 g and an estimated body surface area (BSA)^23^ of 0.007 m^2^, which corresponds to a human adolescent of 13 to 15 years of age.^24^ The ALL-BFM-IC 2009 protocol for childhood leukemia recommends administration of the commercial asparaginase Spectrila at 5000 U/m^2^ every third day, on days 12, 15, 18, 21, 24, 27, 30, 33. Based on the ALL-BFM protocol and the calculated BSA of the animals, the equivalent dose in mice would be approximately 1900 U/kg,^25^ administered on days 12, 15, 18, 21, and 24 from the start of chemotherapy. However, for this study, we adopted a condensed dosing scheme consisting of three concentrated enzyme administrations—1050 and 5250 U/kg on days 0, 14, and 23—without stabilizers or osmolytes, in order to minimize unnecessary stress while enabling the evaluation of potential toxic responses in healthy, immunocompetent mice.

Then, body weight remained unaltered across all groups following multiple ASNase administrations, as all animals showed a steady increase throughout the experiment (Figure 5A). Similarly, body temperature measured after injections on days 0 and 14 (1050 U/kg) remained stable in all groups. However, after the third administration (5250 U/kg), a transient decrease in body temperature was detected in both WT and TM mice compared to controls, 1 h postinjection. Notably, animals recovered baseline temperature values by the end of the 2 h monitoring period (Figure 5A). The lowest recorded temperatures occurred at 0.75 h (45 min) postinjection, reaching 34.3 °C in the WT group and 33.3 °C in the TM group.

**Figure 5:**
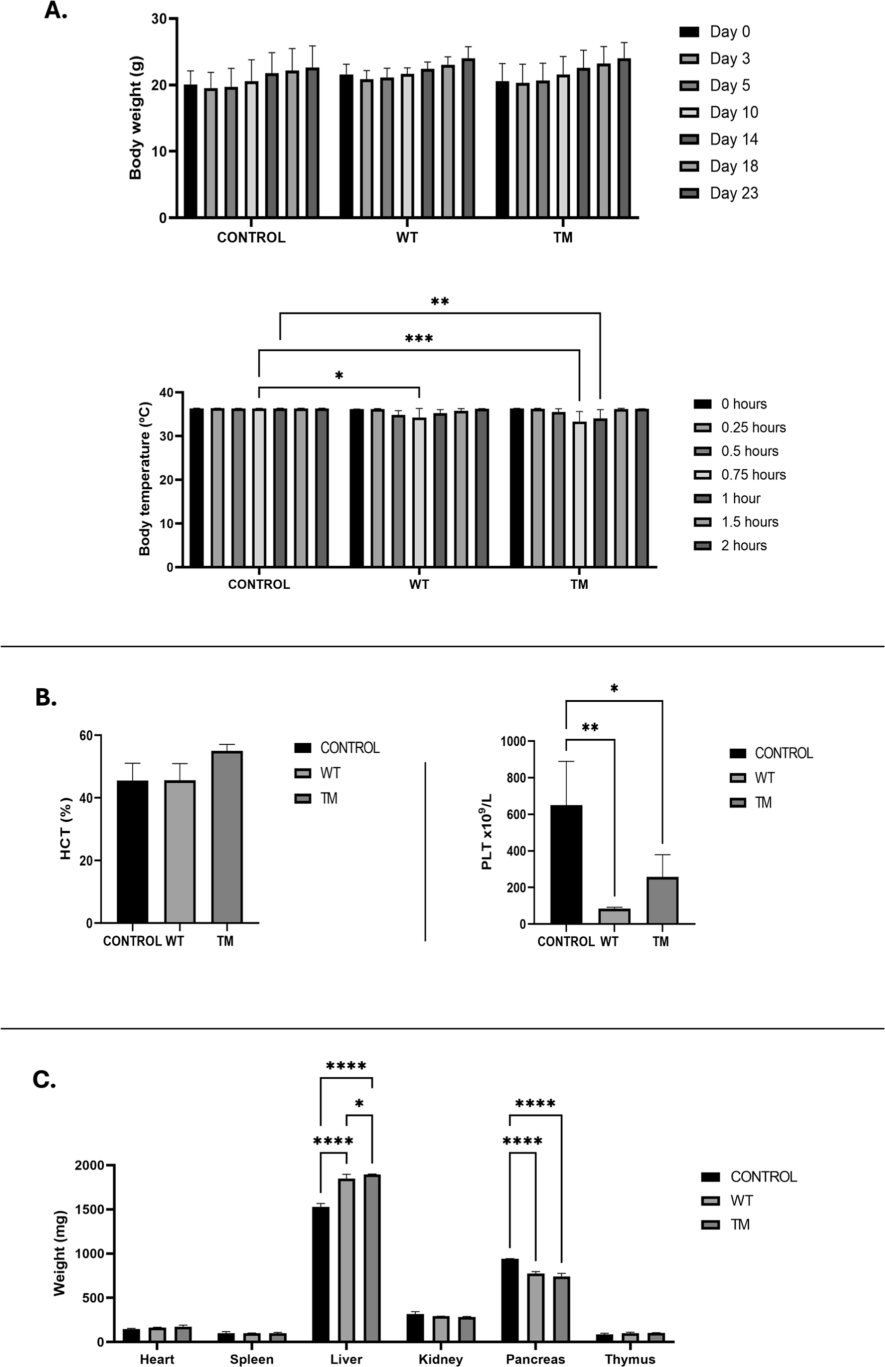
Toxicological responses to multiple ASNase administrations. (A) Body weight monitored throughout the experiment period and body temperature measured up to 2 h after the third dose of each ASNase (*n* = 3 per group). (B) Hematological parameters: hematocrit and platelet count in control, WT, and TM groups (*n* = 3 per group). (C) Organ weights collected after repeated ASNase administrations (*n* = 3 per group). Doses in the toxicological response experiment (*n* = 3 per group). Data are expressed as mean ± SD *p < 0.05; **p < 0.005; ***p < 0.0005; ****p < 0.00005 compared with control (buffer-treated).

Previously, ASNase has been associated with several adverse effects including hypersensitivity reactions, central nervous system dysfunction, hyperglycemia, coagulation abnormalities, liver impairment and pancreatitis.^2,26,27^ Regarding hematological parameters, we observed a condition that could potentially lead to coagulation abnormalities. Platelet (PLT) counts were markedly reduced in both enzyme-treated groups, particularly in WT animals (84.3 × 10^9^/L), followed by TM (258 × 10^9^/L), compared with the control group (650.7 × 10^9^/L) (Figure 5B). Conversely, hematocrit (HCT) values in the TM group slightly exceeded the upper reference limit (55%), although no statistically significant differences were found when compared with the control (45%) or WT (45%) groups (references range: 35% to 45%) (Figure 5B). All other erythrogram and leukogram parameters remained within normal limits.

The reduction in platelet counts may reflect drug-induced immune thrombocytopenia, a condition in which certain drugs or metabolites (e.g., cinchona alkaloids, antibiotics, and nonsteroidal anti-inflammatory drugs) trigger the expression of platelet-reactive antibodies. These antibodies bind to membrane glycoproteins - most notably the αIIbβ3 integrin—leading to platelet destruction, but only in the presence of the soluble drug upon re-exposure.^28^ In this regard, TM exhibited approximately 3-fold lower toxicity compared with WT, suggesting a reduced impact on the coagulation process.

Among organ toxicities, alterations were observed in the liver and pancreas. From all collected organs, only these two showed significant differences compared with the control group. In both enzyme-treated groups, the liver displayed a significant increase in size (1847 mg for WT and 1896 mg for TM) relative to controls (1529 mg). In contrast, the pancreas showed a reduction in weight (775 mg for WT and 740 mg for TM) compared with controls (940 mg) (Figure 5C).

Ruiz-Laraet al. (2024) reported liver impairment in mice treated with multiple doses of single mutant P40S, but not with the S206C variant.^29^ Thus, the increased liver mass observed in TM animals may be attributable to the P40S mutation, possibly due to the accumulation of immune complexes in hepatocytes, since P40S alone has been associated with elevated IgE expression.^29^ Such condition could promote liver steatosis and impair metabolic functions; however, Alex et al. (2015) described this impairment as reversible through exercise, with no long-term complications.^30^

On the other hand, the reduction in pancreas size may reflect tissue degeneration resulting from the loss of healthy pancreatic cells, a condition that could potentially be mitigated by preventive strategies. ASNase-induced acute pancreatic injury has previously been reported in Balb/c mice by Kaya et al. (2014), who demonstrated that l-carnitine supplementation alleviated damage by preserving normal blood levels of biochemical markers such as GSH, MDA, TSA, amylase, glucose, and triglyceride.^27^ Histopathological analysis revealed ASNase-induced toxicity in the exocrine pancreas, particularly affecting acinar cells through vacuolar degeneration or necrosis. Conversely, animals pretreated with l-carnitine were protected, highlighting its antioxidative and tissue-protective effects.^27^

### Immunogenic and Inflammatory Potential

As observed in the toxicological response experiments, body weight and body temperature were not affected by the two ASNase administration doses (1050 U/kg) given on days 0 and 14. All mouse groups showed normal growth rates consistent with age and sex, and no reductions in body temperature were detected on injection days.

In contrast, clear differences emerged in the immunogenic profiles of the enzymes. WT-treated mice displayed a progressive increase in antiasparaginase IgE antibody concentrations from week 2 to week 4 (42 → 57 μg/mL). Conversely, TM-treated mice showed a marked decline in IgE titers, from 56 μg/mL at week 2 to 28 μg/mL at the end of treatment, a significantly lower value compared with WT (Figure 6A). From an immunological standpoint, these results indicate that TM elicits a reduced immunogenic response, characterized by lower anti-ASNase IgE levels and absence of clinical hypersensitivity in vivo. The decline in IgE titers further suggests a lower risk of silent inactivation or hypersensitivity, as reduced immune complex formation limits mast cell and basophil activation, mechanisms that could otherwise impair ASNase efficacy.^9,10,31^ Importantly, IgE–antigen immune complexes are known to be more potent than antigen alone in driving inflammation, promoting cell degranulation, and enhancing antigen presentation by dendritic cells to T and B lymphocytes.^32^

**Figure 6:**
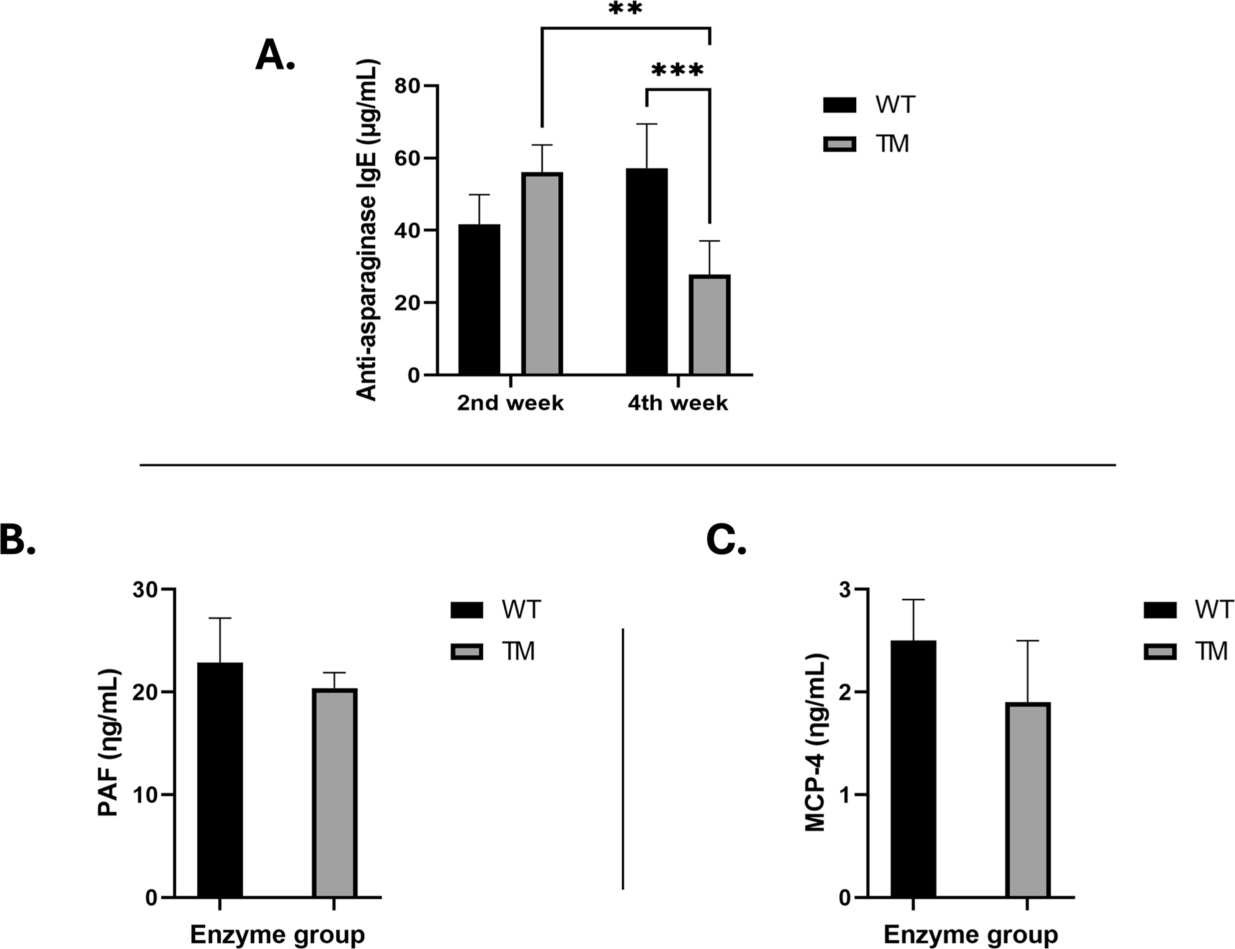
Immunogenic and inflammatory responses to ASNase treatment. (A) Antiasparaginase IgE concentrations in WT- and TM-treated mice at weeks 2 and 4 (*n* = 5 per group). **p < 0.005; ***p < 0.0005 compared with WT. (B) Platelet-activating factor (PAF) and (C) monocyte chemotactic protein-4 (MCP-4) concentrations measured on the final day of the immunogenic/inflammatory assessment (*n* = 5 per group).

To further assess the downstream effects of IgE-mediated immune activation, we analyzed PAF and MCP-4 levels, which reflect products released from basophils/mast cells and monocytes, respectively. Platelet-activating factor (PAF) concentrations were comparable between WT- and TM-treated mice (23 vs 20 ng/mL) (Figure 6B), as were monocyte chemotactic protein-4 (MCP-4) levels (2.5 vs 2.0 ng/mL) (Figure 6C). In sensitized mice, ASNase antigens may bind cell-associated IgE or the high-affinity IgE receptor FcεRI, leading to basophil activation and PAF release.^33^ In mast cells, FcεRI–IgE engagement promotes chemokine release that supports CD4^+^ Th2 responses, partly by enhancing dendritic cell migration to draining lymph nodes and facilitating antigen-specific immunity.^34^ Mast cells and monocytes also contribute to inflammation through chemokines such as MCP-4, which recruit T cells and neutrophils while increasing vascular permeability.^35,36^

In our study, TM-treated animals—consistent with their reduced IgE levels and lower cell degranulation—displayed slightly lower PAF/MCP-4 concentrations compared with WT. This modest reduction may explain the stronger positive feedback on IgE expression observed in WT animals. Importantly, normal white blood cell counts indicated the absence of systemic inflammation in both groups. Thus, despite the platelet alterations detected, TM treatment was associated with milder hematological changes and an overall safer immunological profile than WT.

### Pharmacokinetic Profile

Within the first 2 h after ASNase injection, WT maintained significantly higher enzymatic activity in the bloodstream (8.6 U/mL) compared with TM (6.0 U/mL). Considering an average blood volume of 80 μL per gram of body weight in mice^37^ and an administered dose of 1050 U/kg (equivalent to 13.125 U/mL), WT- and TM-treated mice retained 65.5% and 45.7%, respectively, of their initial relative ASNase activity at this time point.

However, at later time points, TM displayed a clear pharmacokinetic advantage. At 12 h postinjection, TM activity reached 1.04 U/mL (8.0% of initial), compared with only 0.24 U/mL (1.8%) for WT, representing a 4.3-fold increase. At 24 h, TM still maintained 0.15 U/mL (1.2% of initial), while WT had declined to 0.06 U/mL (0.5%), a 2.5-fold difference (Figure 7).

**Figure 7:**
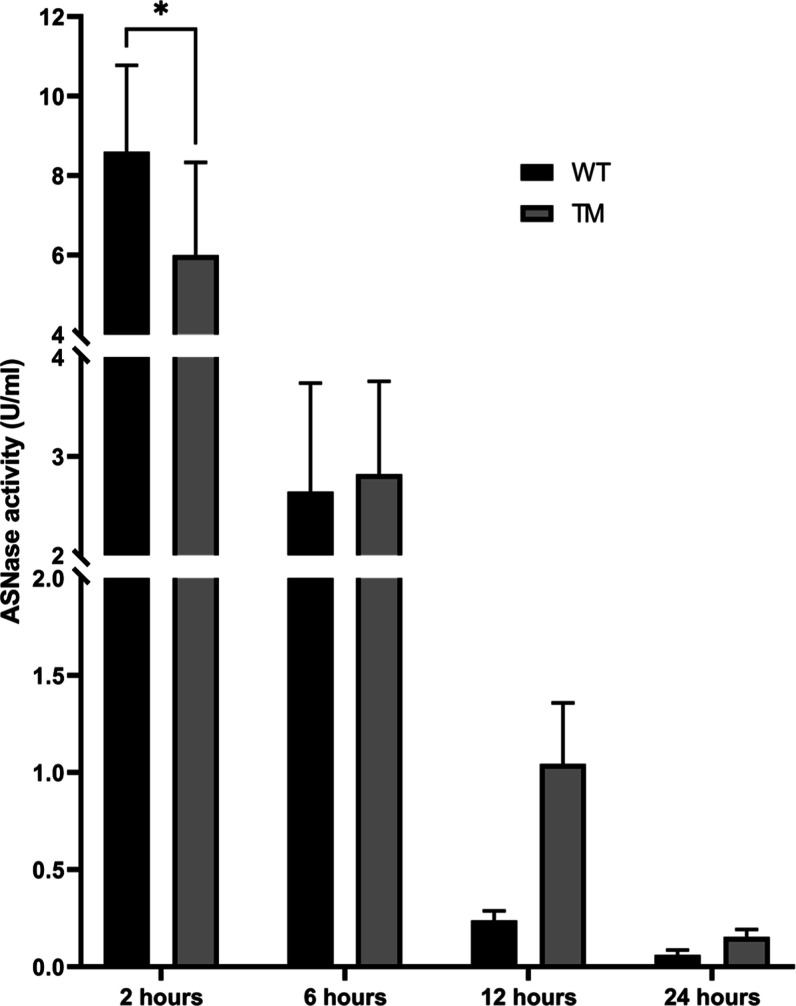
Pharmacokinetic profile of WT and TM ASNase in mice. Enzymatic activity measured in the bloodstream after a single intraperitoneal dose of ASNase (1050 U/kg; *n* = 5 per group). *p < 0.05 indicates statistically significant differences compared with WT.

Overall, pharmacokinetic analysis revealed that TM sustains plasma activity above the therapeutic threshold (>0.02 U/mL) for extended periods, outperforming WT at both 12 and 24 h. This prolonged half-life is likely attributable to reduced proteolytic degradation and diminished immune-mediated clearance—two major limitations of native and even PEGylated ASNases.^16,38^ Remarkably, TM exceeded minimal therapeutic activity threshold by 52-fold at 12 h and 7.5-fold at 24 h, whereas WT reached only 12-fold and 3-fold, respectively.

In summary, the TM ASNase variant developed in this study addresses key limitations of current asparaginase therapies by combining enhanced substrate affinity, improved thermal and proteolytic stability, prolonged in vivo activity, and reduced immunogenicity. These attributes highlight its translational potential for hematology, particularly in the treatment of acute lymphoblastic leukemia (ALL), where rapid enzyme clearance, hypersensitivity reactions, and the need for frequent dosing remain major clinical challenges. Nevertheless, extrapolation to human clinical settings will require validation in additional preclinical models and comprehensive safety assessments. By providing a *biobetter* candidate capable of sustaining asparagine depletion at lower or less frequent doses, our findings support the development of more personalized, safer, and effective treatment strategies for ALL. Furthermore, this work broadens the scope for next-generation enzyme-based biotherapeutics in hematologic malignancies and potentially other diseases.

## Material and Methods

### Enzyme Production

#### Gene and Vector Information

The *ansB* gene (UniProt P00805), codon-optimized for *E. coli* ASNase type II (EC 3.5.1.1), was previously synthesized by GenScript (Piscataway, NJ, USA). Our group generated the double mutant P40S/S206C using error-prone PCR with the GeneMorph II Random Mutagenesis Kit (Agilent Technologies, Santa Clara, CA, USA).^10^ This mutated gene, cloned into the pET15b vector, served as the template for generating an additional asparaginase variant through site-directed mutagenesis (QuikChange Site-Directed Mutagenesis Kit, Agilent Technologies). Using primers Ec_asnB_N24Sfw (GGCGATTCGGCAACCAAAAGCAGCTATACGGTGGGCAAGGTTGG) and Ec_asnB_N24Srev (CCAACCTTGCCCACCGTATAGCTGCTTTTGGTTGCCGAATCGCC), we introduced the N24S substitution. The accuracy of the triple mutant (N24S/P40S/S206C), referred to here as TM, was confirmed by DNA sequencing of the T7-flanked region using the DYEnamic ET Dye Terminator Kit with Thermo Sequenase II DNA Polymerase (Cytiva). The plasmids encoding the WT and TM enzymes were propagated in *E. coli* DH5α and isolated using the QIAprep Spin Miniprep Kit (Qiagen) for storage.

#### Bacterial Transformation and Gene Expression

Electrocompetent *E. coli* BL21 (DE3) cells (Novagen, Merck-Millipore, Burlington, MA, USA) were transformed with 50 ng of pET15b-ansB (WT or N24S/P40S/S206C) using a 2.5 kV pulse on a Gene Pulser II (Bio-Rad). Cells were recovered in 1 mL of fresh lysogeny broth (LB; 10 g/L tryptone, 5 g/L yeast extract, 5 g/L NaCl) at 180 rpm and 37 °C for 1 h. Subsequently, 20 μL aliquots were plated on solid LB medium (supplemented with 2% agar) containing 50 μg/mL carbenicillin (Thermo Fisher Scientific, Carlsbad, CA, USA). Single colonies were cultured overnight at 37 °C and 250 rpm in 400 mL LB. The preinoculum was then diluted into 1 L of fresh medium to an OD_600_ of 0.2. Upon reaching an OD_600_ of 0.7–0.8, protein expression was induced by adding 1 mM isopropyl β-d-1-thiogalactopyranoside (IPTG), and cultures were incubated for 22 h at 37 °C and 200 rpm. Cells were harvested by centrifugation at 4000*g* for 20 min at 4 °C and subjected to osmotic shock to isolate the periplasmic fraction.^10^

#### Protein Purification and Quantification

Osmotic shock, anion exchange chromatography, size-exclusion chromatography, and SDS-PAGE were performed as previously described by Ruiz-Lara et al. (2024).^29^ Protein concentrations were determined by measuring absorbance at 280 nm using a SpectraMax M2 microplate reader (Molecular Devices, CA, USA) equipped with a quartz cuvette, applying the Lambert–Beer law (eq 1):

Equation 1. Lambert–Beer equation used for ASNase quantification

1 $$A_{280nm}=\varepsilon \times l\times c$$

A_280_ nm → absorbance at 280 nm. ε → molar extinction coefficient of WT and TM: 23,505 M^–1^ cm^–1^. l → distance: 1 cm. c → protein concentration (M)

### Enzyme Biochemical Characterization

ASNase and GLNase activities were determined using the Nesslerization reaction, as previously described by Ruiz-Lara et al. (2024).^29^ Specific activity was expressed in international units (U) per milligram of pure enzyme (U/mg), where one unit (U) of ASNase activity is defined as the amount of enzyme required to produce 1 μmol of ammonia per minute at 37 °C and pH 8.8. Statistical analysis was performed using an unpaired *t*-test in Prism 9.0 (GraphPad, La Jolla, CA, USA).

To determine the optimal pH of each ASNase variant (WT and TM), the ASNase specific activity assay described previously was performed using different buffers at a concentration of 50 mM: citric acid (pH 4.0 and 5.0); potassium phosphate (pH 6.0, 7.0, and 8.0); Tris–HCl (pH 9.0); and sodium bicarbonate (pH 10.0 and 11.0). In all reactions, 500 mM NaCl was included to minimize the effects of buffer ionic strength. Assays were conducted in technical triplicate and biological duplicate, and statistical differences were evaluated using two-way ANOVA (α = 0.05) followed by Bonferroni’s multiple comparison test in Prism 9.0 (GraphPad, La Jolla, CA, USA).

Optimal temperature was determined by measuring enzyme-specific activity across a temperature range of 30 to 70 °C, as described above, using the optimal buffer for specific activity previously identified (pH 7.5). Thermostability was subsequently assessed by quantifying enzyme activity at the optimal temperature after incubation for 15 h at 37 °C and 2 h at 50 °C. All assays were performed in triplicate, and statistical differences were evaluated using two-way ANOVA (α = 0.05) followed by Bonferroni’s multiple comparisons test in Prism 9.0 (GraphPad, La Jolla, CA, USA).

The thermal deactivation constant for l-asparaginase was estimated from time-dependent experimental data using eq 2, through linear fitting based on the method adapted from Bento et al. (2017).^39^

Equation 2: Linear Equation for Experimental Quantification of Thermal Deactivation of ASNase

2 $$\ln At=\ln{At}_{0}-K_{d}t$$

At → initial specific activity (U/mg). At_0_→ final specific activity (U/mg). *K*_d_ → thermal deactivation constant (h^–1^). t → time (hours)

The enzyme half-life (t_1_/_2_) was defined as the time required for a 50% reduction in initial specific activity and was calculated using eq 3, based on the deactivation constant (*K*_*d*_) estimated from eq 2.

Equation 3: Equation to determinate the half-life of ASNase enzyme (hours).

3 $$t_{1/2}=\frac{\ln0.5}{{‐K}_{d}}$$

The thermal stability factor (TSF) was calculated as the ratio between the half-life of the TM enzyme and that of the WT enzyme, as shown in eq 4.

Equation 4: Thermal stability factor of TM ASNase enzyme.

4 $$TSF=\frac{t_{1/2}TM}{t_{1/2}WT}$$

The kinetic profiles of the WT and TM enzymes were evaluated spectrophotometrically by monitoring the coupled enzymatic reaction of glutamic-oxaloacetic transaminase (GOT; G2751, Sigma, St. Louis, MO, USA) and malic dehydrogenase (MDH; M2634, Sigma, St. Louis, MO, USA), as described by Munhoz Costa et al. (2022).^40^ Briefly, 80 nM of each ASNase was incubated with 100 mM Tris–HCl buffer (pH 7.4), 0.4 mM α-ketoglutarate, 0.4 mM NADH, 5 U/mL GOT, 5 U/mL MDH, and a range of 2.5–600 μM l-asparagine (Asn). NADH consumption (in μmol) was continuously monitored by absorbance at 340 nm, using the Lambert–Beer law (ε = 6.22 mM^–1^ cm^–1^). The μmol of NADH consumed were plotted versus time, and the slope corresponded to the initial velocity (V_0_, μmol l-aspartate/min). Using the *V*_0_ values and Asn concentrations, *K*_M_ and maximal velocity (*V*_max_) were determined by nonlinear regression in Prism 9.0 (GraphPad, La Jolla, CA, USA). Blank and negative control reactions (without substrate or enzyme, respectively) were included, and the highest background value was subtracted from the experimental absorbance measurements. All assays were performed in technical triplicates and biological duplicates.

Proteolytic resistance of ASNase against human proteases cathepsin B (CTSB) and asparaginyl endopeptidase (AEP) was assessed as previously described by Patel et al. (2009).^38^ Briefly, 1 μL CTSB (1500 U/mg; Sigma-Aldrich) or 0.4 mg/mL AEP (Abcam) was mixed with 2 μg WT or TM enzyme in 50 mM sodium citrate buffer (pH 5.2) to a final volume of 20 μL and incubated for 4 h at 37 °C. Proteolytic resistance was evaluated by measuring residual ASNase activity using the assay described for specific activity. All assays were performed in triplicate, and statistical analysis was conducted using an unpaired *t*-test (α = 0.05) in Prism 9.0 (GraphPad, La Jolla, CA, USA).

### In Silico Analysis

Linear B-cell epitopes were predicted using the Immune Epitope Database and Analysis Resource (IEDB; http://tools.immuneepitope.org/main/) to assess the immunogenic profile of each ASNase based on their amino acid sequences. Predictions were performed with the BepiPred Linear Epitope Prediction tool, applying a threshold of 0.35.

### Nonclinical Tests

#### In Vitro Cytotoxicity Assay

Acute lymphoblastic leukemia cell lines REH and MOLT-4 were obtained from the Rio de Janeiro Cell Bank (RJ, Brazil). Cells were cultured at 37 °C with 5% CO_2_ in RPMI-1640 medium (Vitrocell) supplemented with 10% fetal bovine serum (FBS; Vitrocell), 2 mM glutamine, 10 mM HEPES, 1 mM pyruvate, and 4.5 g/L glucose.

Clear 96-well flat-bottom Nunc microplates (Thermo Scientific, Denmark) were used to seed 5 × 10^3^ cells per well, incubated for 72 h in 150 μL of medium containing seven different concentrations of WT or TM ASNase (0, 0.01, 0.05, 0.1, 0.3, 0.5, and 1 U/mL), as well as a negative control without ASNase (buffer only). Next, 0.5 mg/mL MTT (3-(4,5-dimethylthiazol-2-yl)-2,5-diphenyltetrazolium bromide) was added, followed by incubation for 3 h at 37 °C with 5% CO_2_.

Microplates were centrifuged at 1000*g* for 10 min, the supernatant was discarded, and 200 μL dimethyl sulfoxide (DMSO) was added to each well. Plates were homogenized for 5 min under shaking, and absorbance was measured at 597 nm using a SpectraMax M2 spectrophotometer (Molecular Devices). Absorbance readings from blanks (0 U/mL) were subtracted from the other measurements. The negative control was defined as 100% cell viability, and all results were calculated relative to this control. The assay was performed technical triplicate and biological duplicate, and results were analyzed using an unpaired *t*-test (α = 0.05) in Prism 9.0 (GraphPad, La Jolla, CA, USA).

#### In Vivo Studies

This project was approved by the Ethical Committee for Animal Experimentation of the School of Pharmaceutical Sciences, University of São Paulo (protocol CEUA/FCF 036.2020-P605), for testing the enzymes in male Balb/c SPF mice (6–8 weeks old) following the scheme shown in Figure 8. Animal care was continuously monitored according to the parameters of the Grimace Scale for mice, following guidance from the National Centre for the Replacement, Refinement & Reduction of Animals in Research (NC3Rs).

**Figure 8:**
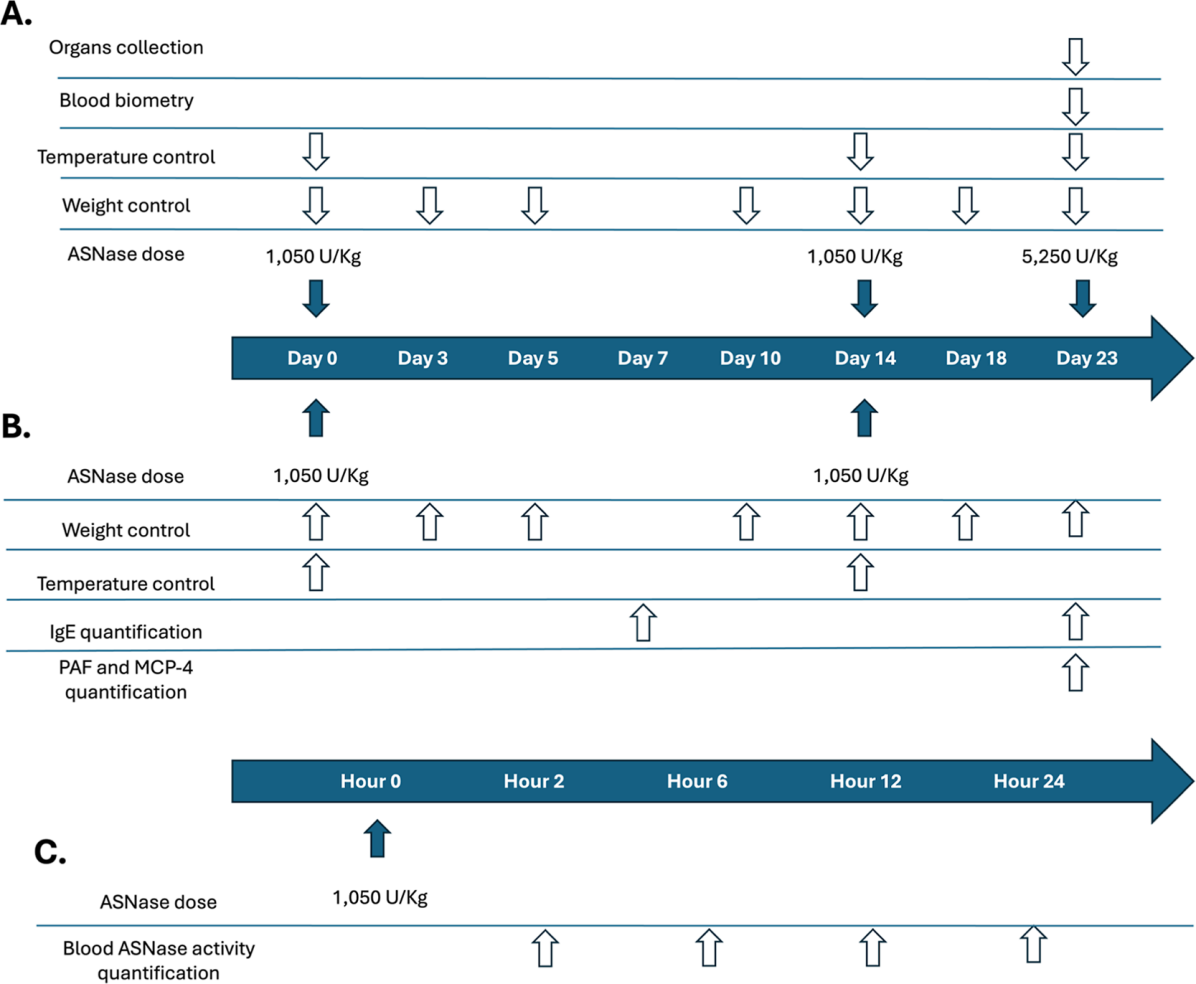
Schematic representation of the independent in vivo experiments performed with WT and TM enzymes. (A) Experimental design for toxicological assessment. (B) Experimental design to evaluate allergenic and inflammatory potential. (C) Experimental design to assess the pharmacokinetics of WT and TM enzymes.

Mice were maintained on a 12 h light/12 h dark cycle with food and water provided ad libitum, at a relative humidity of 45–65% and a temperature of 20–24 °C. ASNase doses were administered intraperitoneally (ip). Three separate experiments were performed to assess toxicological responses, immunogenic and inflammatory potential, and pharmacokinetic profile; for each experiment, animals were divided into three groups: control (buffer only), WT, and TM.

As shown in Figure 8A, for toxicological tests multiple doses of ASNase were administered to each group (three animals per group), with continuous monitoring of body weight and body temperature. In addition, blood parameters and organ samples were evaluated at the experimental end point. Body temperature was measured using an NX-2000 infrared digital thermometer for up to 2 h after enzyme injection. Statistical differences in body weight and temperature were analyzed using two-way ANOVA (α = 0.05) followed by Tukey’s multiple comparison test in Prism 9.0 (GraphPad, La Jolla, CA, USA).

Finally, 2.5 h after the challenge dose of each ASNase, mice were anesthetized with isoflurane, exsanguinated, and blood samples were collected for hematological analysis. A total of 100 μL of whole blood, containing 15 mg/mL EDTA, was analyzed using a BC-2800 Vet Hematological Analyzer (Mindray), with reference values established according to the age of the mice. Statistical differences among groups for hematological parameters were evaluated by one-way ANOVA (α = 0.05) followed by Tukey’s multiple comparison test in Prism 9.0 (GraphPad, La Jolla, CA, USA).

Immediately afterward, the animals were perfused transcardially with 100 mL of ice-cold 1× PBS to remove residual blood, followed by fixation with 200 mL of ice-cold 4% paraformaldehyde (PFA, pH 6.9). The heart, liver, kidneys, thymus, spleen, and pancreas were then harvested, rinsed with 0.9% saline solution, and weighed. Statistical analysis of organ weights was performed in Prism 9.0 (GraphPad, La Jolla, CA, USA) using two-way ANOVA (α = 0.05) followed by Tukey’s multiple comparison test.

In the experiment assessing allergenic and inflammatory potential, molecules such as IgE, platelet-activating factor (PAF), and monocyte chemoattractant protein-4 (MCP-4) were quantified, along with monitoring of body weight and temperature, as shown in Figure 8B (five animals per group). Blood samples were collected on days 7 and 23 in 2 mL tubes containing 15 mg/mL EDTA, centrifuged at 8000*g* at 4 °C for 3 min, and plasma was separated and stored at – 80 °C until analysis. Quantification of specific anti-ASNase IgE (Mouse IgE ELISA Kit, Sigma RAB0799–1KT, USA), PAF (PAF ELISA Kit, CK-bio-16762, EZ Assays, Deerfield Beach, FL, USA), and MCP-4 (CCL13/MCP-4 ELISA Kit, CK-bio-16613, EZ Assays, FL, USA) was performed according to the manufacturers’ instructions. Statistical analysis of IgE levels was performed using two-way ANOVA (α = 0.05) followed by Tukey’s multiple comparison test, while PAF and MCP-4 data were analyzed using an unpaired *t*-test (α = 0.05) in Prism 9.0 (GraphPad, La Jolla, CA, USA).

The final experiment assessed the pharmacokinetic profile of each enzyme after a single dose of 1050 U/kg, with five animals per group (Figure 8C). Blood samples were collected in 2 mL tubes containing 15 mg/mL EDTA, centrifuged for 3 min at 8000*g* and 4 °C; plasma was then separated and stored at −80 °C until analysis. Asparaginase activity was measured as described by Cecconello et al. (2020).^41^ A standard curve (0–2 U/mL) was generated under the same reaction conditions to interpolate absorbance values obtained from the mouse plasma samples. Statistical analysis of asparaginase activity was performed in Prism 9.0 (GraphPad, La Jolla, CA, USA) using two-way ANOVA (α = 0.05) followed by Bonferroni’s multiple comparison test.

### Declaration of Generative AI and AI-Assisted Technologies in the Writing Process

During the preparation of this work, the author(s) used ChatGPT Plus version 5.0 to assist in improving the quality of the language of the human-written text. After using this tool, the author(s) carefully reviewed and edited the content as necessary and assume full responsibility for the final version of the manuscript.

## Abbreviations

AEP: asparaginyl endopeptidase
ALL: acute lymphoblastic leukemia
ANOVA: analysis of variance
Asn: asparagine
ASNase: asparaginase
Balb/c SPF: mouse inbred strain Balb/c specific pathogen-free
BSA: body surface area
CEUA/FCF: Ethical Committee for Animal Experimentation of the School of Pharmaceutical Sciences
CO_2_: carbon dioxide
CTSB: cathepsin B
DMSO: dimethyl sulfoxide
DNA: desoxyribonucleic acid
*E. coli*.: *Escherichia coli*
EcA II: *E. coli* asparaginase type II
EDTA: 2,2′,2″,2‴-(ethane-1,2-diyldinitrilo)­tetraacetic acid
ELISA: enzyme-linked immunosorbent assay
FBS: fetal bovine serum
FDA: food and drug administration
GLNase: glutaminase
GOT: Glutamic-oxaloacetic transaminase
GSH: glutathione
HCT: hematocrit
HEPES: 2-[4-(2-hydroxyethyl)­piperazin-1-yl]­ethanesulfonic acid
iAMP21: Intrachromosomal Amplification of chromosome 21 B-cell precursor acute lymphoblastic leukemia
IgE: immunoglobulin type E
ip: intraperitoneal
IPTG: isopropyl β-d-1-thiogalactopyranoside
*K*_d_: Thermal deactivation constant
*K*_M_: Michaelis–Menten constant
LB: lysogeny broth medium
MCP-4: monocyte chemoattractant protein-4, also known as CCL13
MDA: Malondialdehyde
MDH: malic dehydrogenase
MOLT-4: Human Acute T-Lymphoblastic Leukemia Cell Line
MTT: 3-(4,5-dimethylthiazol-2-yl)-2,5-diphenyltetrazolium bromide
NaCl: Sodium chloride
NADH: Nicotinamide Adenine Dinucleotide (reduced)
NC3Rs: National Centre for the Replacement, Refinement & Reduction of Animals in Research
PAF: Platelet-activating factor
PBS: phosphate-buffered saline
PEG: Polyethylene glycol
pH: Potential of hydrogen
Ph+: philadelphia positive leukemia
PLT: platelet
REH: B-cell precursor acute lymphoblastic leukemia cell line
RPMI: Roswell Park Memorial Institute cell culture medium
*t*_1_/_2_: half-life
TM: Triple mutant
Tris–HCl: tris­(hydroxymethyl)­aminomethane hydrochloride
TSA: total serum sialic acid
TSF: thermal stability factor
V_0_: initial velocity
*V*_max_: maximal velocity
WT: wild-type
